# Development of the *in vitro* Cecal Chicken ALIMEntary tRact mOdel-2 to Study Microbiota Composition and Function

**DOI:** 10.3389/fmicb.2021.726447

**Published:** 2021-10-11

**Authors:** Miriam J. Oost, Francisca C. Velkers, Aletta D. Kraneveld, Koen Venema

**Affiliations:** ^1^Centre for Healthy Eating and Food Innovation, Faculty of Science and Engineering, Maastricht University-Campus Venlo, Venlo, Netherlands; ^2^Division Farm Animal Health, Department Population Health Sciences, Faculty of Veterinary Medicine, Utrecht University, Utrecht, Netherlands; ^3^Division of Pharmacology, Utrecht Institute for Pharmaceutical Sciences, Faculty of Science, Utrecht University, Utrecht, Netherlands; ^4^Centre for Healthy Eating and Food Innovation, Department of Human Biology, Faculty of Health, Medicine and Life Sciences, Maastricht University, Venlo, Netherlands

**Keywords:** *in vitro* cecal model, chicken, CALIMERO-2, bacterial composition, SIEM

## Abstract

The digestive system of the chicken plays an important role in metabolism, immunity, and chicken health and production performance. The chicken ceca harbor a diverse microbial community and play a crucial role in the microbial fermentation and production of energy-rich short-chain fatty acids (SCFA). For humans, dogs, and piglets *in vitro* digestive system models have been developed and are used to study the microbiota composition and metabolism after intervention studies. For chickens, most research on the cecal microbiota has been performed in *in vivo* experiments or in static *in vitro* models that may not accurately resemble the *in vivo* situations. This paper introduces an optimized digestive system model that simulates the conditions in the ceca of the chicken, i.e., the Chicken ALIMEntary tRact mOdel-2 (CALIMERO-2). The system is based on the well-validated TNO *in vitro* model of the colon-2 (TIM-2) and is the first dynamic *in vitro* digestion model for chickens species. To validate this model, the pH, temperature, and different types of microbial feeding were compared and analyzed, to best mimic the conditions in the chicken ceca. The bacterial composition, as well as the metabolite production at 72 h, showed no significant difference between the different microbial feedings. Moreover, we compared the CALIMERO-2 digestive samples to the original inoculum and found some significant shifts in bacterial composition after the fermentation started. Over time the bacterial diversity increased and became more similar to the original inoculum. We can conclude that CALIMERO-2 is reproducible and can be used as a digestive system model for the chicken ceca, in which the microbial composition and activity can be maintained and shows similar results to the *in vivo* cecum. CALIMERO-2 can be used to study effects on composition and activity of the chicken cecum microbiota in response to in-feed interventions.

## Introduction

The digestive system of the chicken plays a pivotal role in metabolism, immunity, and therewith in the health and production performance (Gong et al., 2002; Round and Mazmanian, 2009; Xiao et al., 2017; Borda-Molina et al., 2018). The gastrointestinal tract (GIT) of poultry differs from the GIT of mammals in many ways, including a shorter size relative to body length, and the size and role of the ceca (Svihus, 2014; Yadav and Jha, 2019). The ceca play an important role in the GIT of poultry, because of active microbial fermentation and the production of energy-rich short-chain fatty acids (SCFA) (Ocejo et al., 2019; Liao et al., 2020). The avian ceca harbor a diverse microbial community that is dominated by strict anaerobic bacteria (Zhu et al., 2002). Many factors can influence the microbial composition (Kers et al., 2018). Some perturbations can induce a shift in the intestinal microbiota composition and can lead, for instance, to the enteric disease necrotic enteritis (Antonissen et al., 2016; Lu et al., 2020). Many studies have aimed to optimize the gut microbiota of mammals as well as that of chickens with dietary interventions. For example. previous studies have shown that prebiotics can stimulate the growth of beneficial endogenous microbes by providing nutrients to beneficial bacteria (Gibson et al., 2004; Adhikari et al., 2020), and can lead to better growth and health of the chickens (Chambers and Gong, 2011). By stimulating beneficial bacteria, relative abundance of harmful bacteria like *Clostridium perfringens* can be reduced. Most of the current research on the microbiota in the chicken GIT has been performed in *in vivo* experiments or field studies (De Carvalho et al., 2021). *In vivo* experiments have many downsides, including that these methods are invasive for the animals or in case of non-invasive methods like cloacal swabs, these might not completely represent the composition of the ceca (Minekus et al., 1999; Gibson et al., 2004). Therefore, there is a need for *in vitro* digestive system models to study the behavior of microbiota, with high predictive value for *in vivo* animal trials, to gain in-depth knowledge of the effect and possible mechanisms of action of dietary interventions on the chicken cecal microbiota.

This paper introduces CALIMERO-2, which is an acronym for Chicken ALIMEntary tRact mOdel-2, based on the validated, dynamic, computer-controlled TNO intestinal model of the colon (TIM-2) (Minekus et al., 1999). CALIMERO-2 mimics the cecum of a chicken and can be used to evaluate the effects of feed additives, and other compounds on the microbial composition and activity over time. Here, the dynamic digestive system model was developed and optimized for the chicken species. The effect of different types of microbial feeding was analyzed and compared, to best mimic the chickens’ diet and support the growth of the chicken microbiota. Moreover, bacterial composition in the *in vitro* digestive system model was compared to the original cecal inoculum, to investigate the resemblance with the *in vivo* situation.

## Materials and Methods

### The Chicken ALIMEntary tRact mOdel-2

**FIGURE 1:**
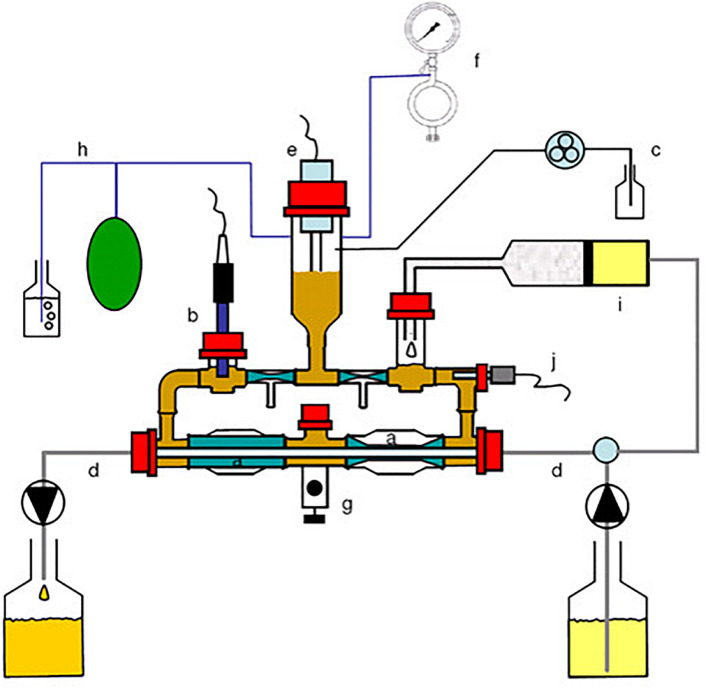
Schematic representation of Chicken ALIMEntary tRact mOdel-2 (CALIMERO-2). a = Peristaltic compartments with a dialysis membrane inside; b = pH sensor; c = NaOH inlet; d = dialysate system; e = level sensor; f = gaseous N_2_ inlet; g = sampling port; h = gas outlet; i = feeding syringe; j = temperature sensor.

The Chicken ALIMEntary tRact mOdel-2 (CALIMERO-2; Figure 1) simulates the ceca of the chicken and is based on the same concept as TIM-2 described by Minekus et al. (1999) and Venema (2015). Briefly, CALIMERO-2 consists of four identical independent units that can be run in parallel. Each unit has four interconnected glass units, with a flexible wall inside. The volume of the lumen is approximately 150 ml. The temperature and the pH are regulated in the system to mimic the body temperature, which for broiler chickens is 41°C at a pH of 6.6 (Van Der Wielen et al., 2000; Mabelebele et al., 2014). The temperature is regulated by pumping water into the space between the glass jacket and the flexible wall [Figure 1 (j)]. Additionally, the water pressure is changed constantly to create peristaltic movements similar to those in the gut [Figure 1 (a) and Supplementary Video 1]. The pH is constantly measured by pH electrodes [Figure 1 (b)] in the system and maintained by adding 2M sodium hydroxide when necessary [Figure 1 (c)]. By flushing the system with nitrogen gas [Figure 1 (f)], the model is kept anaerobic. Moreover, the metabolites produced by the microbiota are continuously filtered out of the lumen by making use of a unique semi-permeable membrane that functions as a dialysis system [Figure 1 (a)]. This dialysate is continuously collected and can be sampled for microbial metabolites [Figure 1 (d)]. By making use of such a dialysis system, the physiological concentrations of, e.g., short-chain fatty acids (SCFA) are maintained and there is no accumulation of these small molecules, which would otherwise lead to inhibition or death of the microbiota within a matter of hours (Venema, 2015). The system was inoculated with a standardized anaerobic cecal microbiota of broiler chickens [Figure 1 (g)], obtained as described below. Furthermore, the microbiota was fed with microbial feedings as described in subsequent sections [Figure 1 (i)].

### Collection of Cecal Samples and Standardization

The cecal content was obtained from slaughterhouse van der Linden Poultry products B.V. (Beringe, Netherlands), where broiler chickens (Ross 308) were brought from local chicken farms. The broiler chickens were fed a coccidiostat-free diet and were not treated with antibiotics the days before slaughter. The birds were stunned, debled and the ceca were removed within 1 h after killing and placed in sterile plastic bags containing an anaerocult^®^ strip (AnaeroGenTM, Cambridge, United Kingdom) and transported on ice where the ceca were processed immediately after arrival, within 1 h after collection of the ceca. In the laboratory, the cecal content was removed and pooled under strictly anaerobic conditions in an anaerobic cabinet (Sheldon Lab –Bactron IV, Gomelius, OR, United States). A total amount of 945 g was 1:1 diluted with dialysis liquid (content per liter: 2.5 g K_2_HPO_4_⋅3H_2_O, 4.5 g NaCl, 0.005 g FeSO_4_⋅7H_2_O, 0.5 g MgSO_4_⋅7H_2_O, 0.45 g CaCl_2_⋅2H_2_O, 0.05 g ox bile, and 0.4 g cysteine hydrochloride, plus 1 ml of vitamin mixture [see next section]) and as a cryo-protective agent, 15% (w/v) glycerol was added. The cecal samples were aliquoted (35ml), snap-frozen in liquid nitrogen, and stored at −80°C.

### Microbial Feeding

To compare microbial composition and activity in CALIMERO-2 to the *in vivo* situation, different feeding types were tested, namely Standard Ileal Effluent Media (SIEM), which is standard for experiments with human microbiota (Maathuis et al., 2009), modified SIEM-I and modified SIEM-II (Supplementary Table 1). We tried to mimic the chickens’ diet in the modified microbial feedings, by replacing arabinogalactan with soy-based arabinoxylan. Furthermore, the potato starch was replaced by wheat and maize starch, since the diet of broiler chickens is composed mainly of soy, maize, and wheat (Attia et al., 2021). Standard Ileal Effluent Media was prepared as described by De Souza et al. (2014) with the following compounds (g L^–1^): 9 citrus peel pectin, 9 beechwood xylan, 9 larch arabinogalactan, 9 potato amylopectin, 74.6 potato starch, 31.5 Tween 80, 43.7 casein, 0.7 ox-bile, 43.7 bactopepton, 4.7 K_2_HPO_4_.3H_2_O, 0.009 FeSO_4_.7H_2_O, 8.4 NaCl, 0.8 CaCl_2_.2H_2_O, 0.7 MgSO_4_.7H_2_O, 0.02 hemin, and 0.3 cysteine⋅HCl, plus 1.5 mL of a vitamin mixture containing (mg L^–1^): 1 menadione, 0.5 vitamin B12, 2 D-biotin, 10 pantothenate, 5 p-aminobenzoic, 4 thiamine, and 5 nicotinamide acid. The pH was adjusted to 6.6 to mimic the chicken ceca and 60 ml/day was administered. Modified SIEM-I was adjusted to mimic chicken feed by replacing citrus peel pectin and larch arabinogalactan with soybean rhamnogalacturonan (9) and raffinose (9). Furthermore, the potato starch component was replaced by 80% wheat and 20% maize starch. Modified SIEM-II had the same components as modified SIEM-I, with additionally 9 g L^–1^ arabinoxylan (Bioactor, Maastricht, Netherlands), oat beta-glucan, and konjac glucomannan and an adjusted composition of starch, namely 50% maize and 50% wheat. Standard SIEM and vitamin mix were purchased from Tritium microbiology (Eindhoven, Netherlands).

### Experimental Setup

Two independent experiments in CALIMERO-2 were done, each using four independent fermentation units which were run simultaneously (Supplementary Figure 1A). In each experiment, two fermentation units included SIEM as control and the other two fermentation units contained either Modified SIEM-I or the Modified SIEM-II. Each experiment started with inoculation of the system with 60 ml of the standardized cecal microbiota, to which 90 ml of pre-reduced dialysis liquid was added. In both experiments, the same batch of inoculum was used. There were five sample time points (t-16 h (time of inoculation), 0 h (after overnight adaptation), 24 h, 48 h, and 72 h) from lumen and dialysate to analyze the microbial composition (lumen) and metabolite composition (lumen and dialysate) over time (Supplementary Figure 1B). All samples were snap-frozen in liquid nitrogen and stored at -80°C until further analysis. After 24 and 48 h, a total volume of 25 ml of lumen sample was removed from the system to simulate passage of chyme to the chicken large intestine (Maathuis et al., 2009).

### Microbial DNA Extraction

DNA was extracted from 250 μl of the lumen samples taken during the CALIMERO-2 experiments using 1000 μl InhibitEx buffer (Qiagen, Venlo, Netherlands). The sample was transferred to a Precyllus tube containing 0.5 mm microbeads and treated in a bead beater (Precellys 24, Bertin technologies, Montigny-le-Bretonneux, France) at a speed of 6000 Hz for 3 × 30 s, with cooling on ice between steps. Afterward, the sample was incubated at 95°C for 7 min and centrifuged (Rotina 420 R, Hettich Benelux B.V. Netherlands), at 13500 g for 1 min to pellet stool particles and cell wall fragments. From this point, the QIAamp DNA stool Mini kit (Qiagen) was used following the manufacturer’s protocol from step 4 onward, with some adjustments. Briefly, 30 μl of proteinase K was added to a 1.5 ml microcentrifuge tube and 400 μl of the supernatant of the sample was added together with 400 μl of Buffer AL and vortexed before the sample was incubated at 70°C for 10 min. After incubation, 400 μl of ethanol (96%–100%) was added and the volume was transferred to a QIAamp spin column in two steps and centrifuged at 13500 g for 1 min. Next, 500 μl of AW1 buffer was added and centrifuged at 13500g for 1 min, followed by addition of 500 μl of AW2 buffer and centrifugation for 3 min. To elute the DNA, the QIAamp spin column was placed into a new microcentrifuge tube and 100 μl of ATE buffer was added, incubated for 3 min at room temperature, and centrifuged at 13500g for 1 min. To quantify the DNA concentration Qubit dsDNA HS Assay kit was used and the DNA was measured using a Qubit 3.0 Fluorometer (Invitrogen, Landsmeer, Netherlands) and stored at −20°C until further use.

### Bacterial Composition

To study the composition of the bacteria during the experimental phase, the composition of the bacteria was evaluated by 16S rRNA gene sequencing using Illumina Miseq (Illumina, San Diego, CA, United States). 16S rRNA gene amplicon libraries of the V3-V4 region were generated following the 16S Metagenomic Sequencing Library preparation manual of Illumina Miseq systems using the Nextera XT kit, using a 2-step PCR. Briefly, in the first step, 10–25 ng genomic DNA was used as template for the first PCR with a total volume of 50 μl using the 341F (5′-CCTACGGGNGGCWGCAG-3′) and 785R (5′-GACTACHVGGGTATCTAATCC-3′) primers appended with Illumina adaptor sequences. PCR products were purified (QIAquick PCR Purification Kit) and the size of the PCR products was checked on a Fragment analyzer (Advanced Analytical, Ankeny, United States) and quantified by fluorometric analysis (Qubit^TM^ dsDNA HS Assay Kit). Purified PCR products were used for the second PCR in combination with sample-specific barcoded primers (Nextera XT index kit, Illumina). Subsequently, PCR products were purified, checked on a Fragment analyzer and quantified, followed by equimolar multiplexing, clustering, and sequencing on an Illumina MiSeq with the paired-end (2x) 300 bp protocol and indexing. A mock community was run along with the samples to guarantee sequence quality.

### Short-Chain Fatty Acids, Branched-Chain Fatty Acids, and Organic Acids Quantification in Lumen and Dialysate Samples

To quantify the SCFA (acetate, propionate, and butyrate), branched-chain fatty acids BCFA (iso-butyrate and iso-valerate), and other organic acids (succinate, formate, lactate, valerate, and caproate) in the samples from the lumen and dialysate, ion exclusion chromatography (IEC) was performed by Brightlabs (Venlo, Netherlands). Briefly, an 883 Ion Chromatograph was used (IC; Metrohm, Switzerland), with a Transgenomic IC Sep ICE-ION-300 column (30 cm length, 7.8 mm diameter, and 7 μm particles) and a MetroSep RP2 Guard. The mobile phase consisted of 1.5 mM aqueous sulfuric acid and the column had a flow rate of 0.4 ml min^–1^ and a temperature of 65°C. The organic acids were detected using suppressed conductivity detection. Samples were centrifuged at 13500 g for 10 min, and the clear supernatant was filtered through a 0.45 μm PFTE filter and diluted with mobile phase (for lumen 1:5, for dialysate 1:2). Ten μl were loaded on the column by an autosampler 730 (Metrohm). Molecules were eluted according to their pKa.

### Bioinformatics Analysis

Microbiota bioinformatics was performed with QIIME2 2019.4 (Bolyen, 2019). Briefly, the raw sequencing data were demultiplexed, quality filtered, and denoised by using the q2-demux plugin and DADA2 (Callahan et al., 2016). In the DADA2 step, the first 9 bases were trimmed off and for the forward reads there was a truncation at 290 base pairs and for the reverse reads, this was at 280 base pairs. Taxonomy was assigned using the SILVA 128 16Sr RNA gene reference database. Further analysis was continued with the packages *microbiome, vegan and phyloseq* after the qza files were converted to phyloseq object with the *qiime2R* package (Bisanz, 2018).

### Statistical Methods

Shannon diversity, inverse Simpson, Gini-Simpson, Fisher, and coverage were calculated to define microbial alpha diversity for each sample by making use of the *phyloseq* and microbiome R packages. Differences in alpha diversity were tested with a Kruskal-Wallis test, and pairwise comparisons were tested using a Wilcoxon rank-sum test and corrected for multiple testing with Benjamini-Hochberg in the open-source software package STAMP v2.1.3 (Parks et al., 2014). For the statistical analysis of the beta diversity for the different feeding types and comparison with the original inoculum, permutational multivariate analysis of variance (PERMANOVA) (Anderson, 2017) was performed. Significance of the SCFA, BCFA and organic acid concentrations among different feeding groups were analyzed by Kruskal-Wallis Rank sum test followed by corrections for multiple testing with the Benjamini-Hochberg method in R. All analyses were done in R version 3.6.2 (R Core Team, 2020).

## Results

### Bacterial Alpha- and Beta Diversity

**FIGURE 2:**
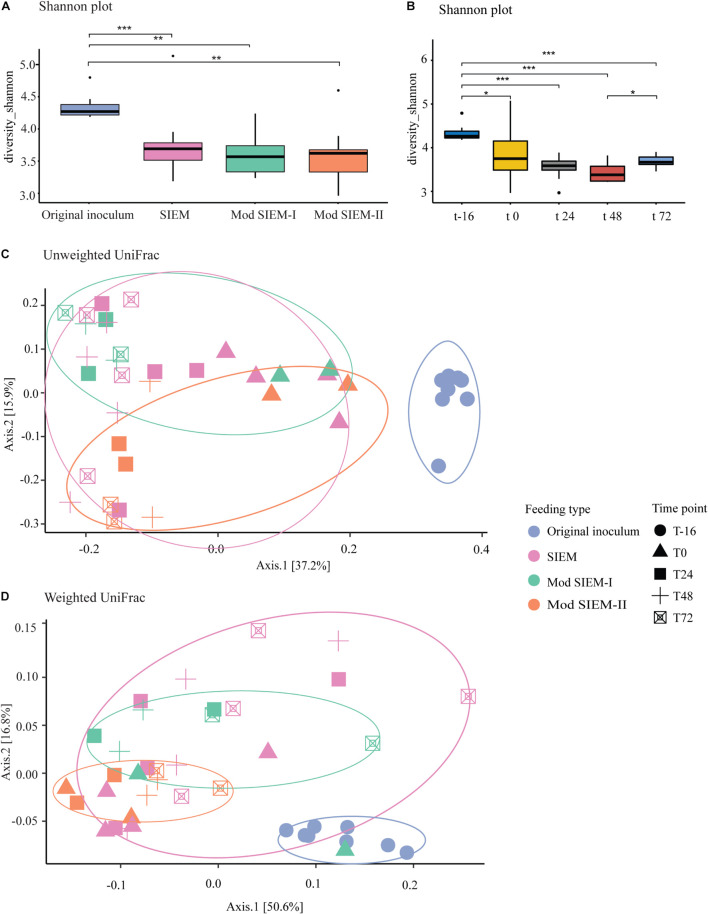
Bacterial diversity. **(A)**. For the alpha diversity, Shannon indexes were calculated to verify the abundance and evenness of the species present in the of Chicken ALIMEntary tRact mOdel-2 (CALIMERO-2) samples. Data are presented as mean (*n* = 2) ± sd. Significant difference is shown between original inoculum and SIEM (*p* < 0.001) and original inoculum and modified SIEM-I and II (*p* < 0.05). **(B)**. Shannon index for the CALIMERO-2 samples taken at different timepoints. The beta diversity is represented as principal coordinates analysis (PCoA) using the unweighted UniFrac **(C)** or the weighted UniFrac **(D)** for the cecal microbiota of chickens form the CALIMERO-2 model.

The bacterial composition of the lumen samples from runs with the different types of microbial feeding, i.e., SIEM, Modified SIEM-I, and Modified SIEM-II, which served as microbial growth medium, were analyzed and compared to the original inoculum, i.e., the pooled and standardized sample before inoculation into the system at time point -16 h. The different sampling time points (0 h, 24 h, 48 h, 72 h) during the fermentation runs in CALIMERO-2 and the original inoculum were also compared. For these samples, the Shannon diversity, to assess the bacterial alpha diversity within a community, was calculated. A significant difference was found between all the feeding types compared to the original inoculum based on the Shannon index (Figure 2A; SIEM: *P* < 0.0001, Mod SIEM-I: *P* < 0.001, Mod SIEM-II: *P* < 0.001), indicating a lower diversity in the *in vitro* digestive system samples, compared to the original inoculum. Between the different feeding types, no significant differences were found. The effect of the fermentation process on the alpha diversity over time was examined using the Shannon index, a significant difference was demonstrated between the different time points and the original inoculum at time point -16 h (Figure 2B; 0 h: *P* < 0.0001, 24 h: *P* < 0.0001, 48 h: *P* < 0.0001 and 72 h: *P* < 0.001). There was also a significant difference between time points 48 h and 72 h (*P* < 0.01) (Figure 2B). Other alpha diversity measures (Inverse Simpson, Gini-Simpson, Fisher, and coverage) are provided in Supplementary Figure 2 and showed the same trend as the Shannon index.

The beta diversity distance matrices weighted and unweighted UniFrac were examined to determine the variance between the different feeding groups. The unweighted UniFrac demonstrate an overlap of clusters of SIEM and modified SIEM-I. Modified SIEM-II and the original inoculum both show a shift in clusters compared to SIEM and modified SIEM-I (*P* < 0.001) (Figure 2C). The principal coordinate plot of the weighted UniFrac show no clustering associated with the different feeding groups, whereas a significant difference was observed between the original inoculum and the different feeding groups (*P* < 0.05) (Figure 2D).

### Taxonomic Analysis

**FIGURE 3:**
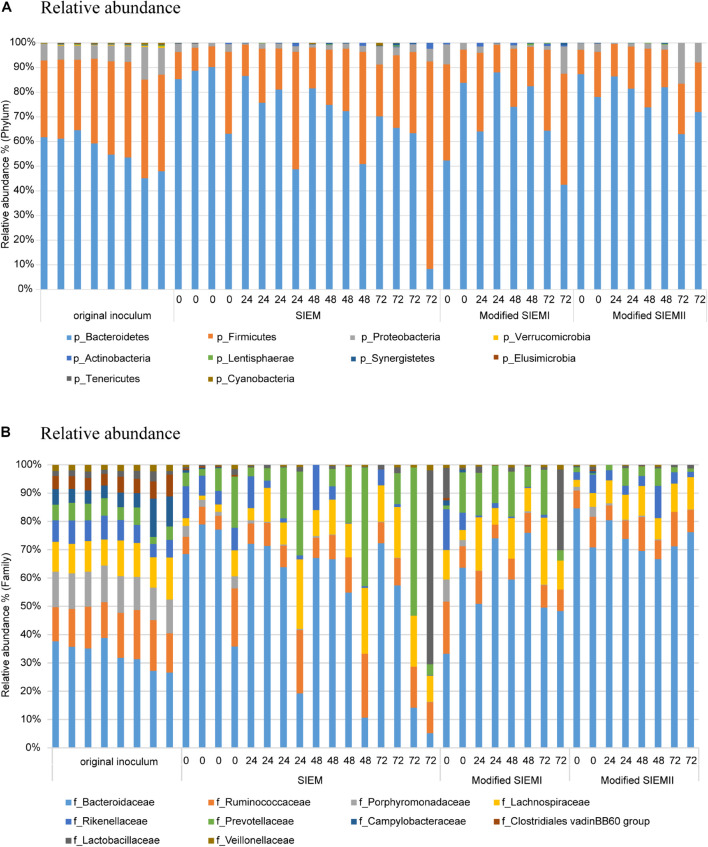
Bacterial composition. Relative abundance of bacterial phyla **(A)** and families **(B)** in Chicken ALIMEntary tRact mOdel-2 samples using different feeding type at different time points compared to the original inoculum.

To assess the effect of feeding types on bacterial composition, the community was analyzed at the taxonomic rankings of phylum and family levels (Figures 3A,B, respectively). The samples obtained with the different feeding types were compared to each other and the original inoculum. The taxonomic profiles at phylum level showed that the dominant populations were *Bacteroidetes* (56%), *Firmicutes* (35%), and *Proteobacteria* (7.6%) in all of the samples. The relative abundance (RA) of the phyla *Verrucomicrobia* (*P* < 0.001), *Cyanobacteria* (*P* < 0.01), and *Tenericutes* (*P* < 0.01) were significantly higher in abundance in the original inoculum samples, compared to the CALIMERO-2 samples (Figure 3A). However, the bacterial composition was not significantly affected by the adjusted microbial feedings compared to the standard medium (SIEM). Moreover, the bacterial composition showed no significant differences between the different time points, within the feeding type groups. For SIEM there seem to be some individual differences, however, most of the samples within the group show similar profiles, and suggest that CALIMERO-2 can be seen as a reproducible system.

Within the phylum *Bacteroidetes*, the family *Porphyromonadaceae* was significantly higher in relative abundance in the original inoculum compared to the samples obtained from the CALIMERO-2 (*P* < 0.01). Moreover, the families *Clostridiales vadin BB60 group, Erysopelotrichaceae*, and *Veillonellaceae*, which belong to the phylum *Firmicutes*, were all significantly higher in the original inoculum, compared to the other samples (*P* < 0.01). Furthermore, *Campylobactereaceae* also showed a significant decrease in the CALIMERO-2 fermented samples, compared to the original inoculum (*P* < 0.001).

### Production of Short-Chain Fatty Acids, Branched-Chain Fatty Acids, and Organic Acids

**FIGURE 4:**
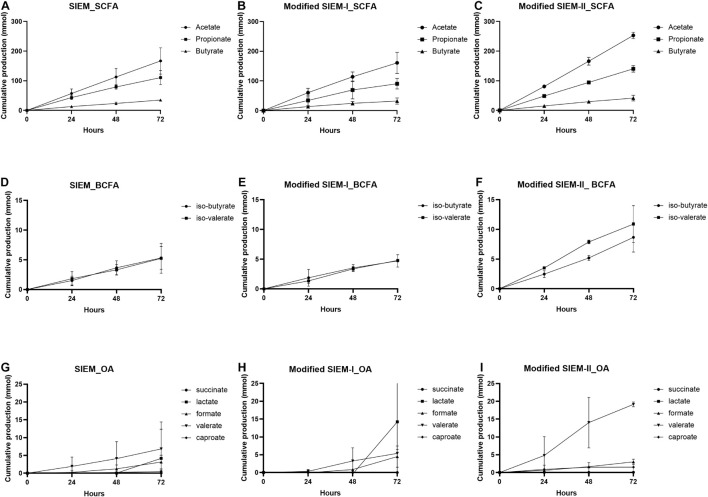
Production of microbial metabolites over time. Cumulative production (in mmol) over time of short chain fatty acids (SCFA), branched chain fatty acids (BCFA) and other organic acids (OA). SIEM **(A,D,G)**, modified SIEM-I **(B,E,H)** and, modified SIEM-II **(C,F,I)** Data are presented as mean (*n* = 2) ± sd.

To characterize the fermentation concerning microbial activity, the cumulative total production of SCFA, BCFA, and other organic acids, representing the sum of metabolites that were present in the lumen and the dialysate, were measured over time and shown in Figure 4. Overall, the production of SCFA was very similar between the tested microbial feedings and no significant difference was observed. For all the samples, the acetate production was the highest, followed by propionate and butyrate that showed the lowest cumulative production (Figures 4A–C). The BCFA production showed also no significant difference between the different feeding types (Figures 4D–F). When comparing the production of BCFA to SCFA, the amount BCFA produced was much lower than that of SCFA. The production of the other organic acids, succinate and caproate, was negligibly small, but the lactate, valerate and formate production was evident in time, but much lower than the SCFA production. The production over time of the other organic acids shows a delay in production compared to SCFA and BCFA. The other organic acid production started between 24 and 48 h (valerate) or even between 48 and 72 h of fermentation, whereas for SCFA and BCFA production is already evident at the start and 24 h of fermentation (Figures 4 G–I).

## Discussion

Prior work has documented that microbial composition can be influenced by many factors, for example by the addition of prebiotics to the diet. Testing the effect of different types of manipulations of the microbial composition, for instance with feed additives are important for further understanding of the modes of action and expected effects on improving animal intestinal health and performance (Round and Mazmanian, 2009; Scott et al., 2013; Yadav and Jha, 2019; Lu et al., 2020). Research on human and pig microbiota can be performed in *in vitro* digestive system models, for example in the well-established and predictive TIM-2 and SLIM (Swine Large Intestinal Model) systems. These two models have been compared and validated to *in vivo* conditions (Minekus et al., 1999; Venema et al., 2000, 2003; Maathuis et al., 2009; Venema, 2015; Long et al., 2020). To study the effect of dietary substrates on the chicken gut microbiota, experiments have been performed *in vivo*, or data were obtained from a static fermentation model or the SHIME model (Simulator of the Human Intestinal Microbial Ecosystem) (Van Der Wielen et al., 2004; De Maesschalck et al., 2015). So far, there is no validated, advanced dynamic *in vitro* digestion system model known for chickens.

In this study, we established and optimized a dynamic *in vitro* cecal digestive system model for chickens to mimic the *in vivo* situation, which is based on the TIM-2 model. For the chicken model, we changed the pH and the body temperature, respectively, to 6.6 and 41°C (Mabelebele et al., 2014). In addition, we compared two adjusted microbial feedings to the standard microbial feeding SIEM used for the human microbiota. In previous experiments with SLIM, we showed the need to optimize the composition of SIEM to allow the microbiota to stay close to the original pig inoculum (Long et al., 2020). In our experiments here, we show that SIEM itself performs well in this system, and changing the medium composition of SIEM does not lead to a better representation of the microbiota composition.

To validate CALIMERO-2, the original inoculum was used to represent the *in vivo* situation and CALIMERO-2 samples were compared to it. The taxonomic profile of the bacterial composition of the original inoculum showed that the phyla *Bacteroidetes*, together with *Firmicutes* and *Proteobacteria*, were most abundant, which is consistent with earlier research of Corrigan et al. (2015); Oakley and Kogut (2016). In contrast to these previous studies, the relative abundance of the *Firmicutes* and *Bacteroidetes* relative to each other was reversed in our study. This different ratio of *Firmicutes* and *Bacteroidetes* might have been caused, amongst others, by differences in the type of feed the chickens received, housing conditions, the genetic background of the chickens, or different processing of the samples before analysis (Rajilić-Stojanović et al., 2010; Corrigan et al., 2015; Kers et al., 2019).

Although previous research suggests that diet affects bacterial composition and its metabolite production, the changes we applied did not result in significant differences between the feeding types in bacterial composition and metabolite production (Long et al., 2020), indicating that the unmodified SIEM can be used in CALIMERO-2. To illustrate this, the taxonomic profile was examined. For all feeding types, *Bacteroidetes, Firmicutes*, and *Proteobacteria* remained most abundant over time, some of the other bacterial phyla, such as *Campylobacter*, were significantly decreased over time compared to the original inoculum. The reduction of these microaerophilic taxa might be caused by the lack of oxygen in the strict anaerobic environment in the system. Since the majority of cecal colonizers are strict anaerobes (Rychlik, 2020), we maintained strict anaerobic conditions in CALIMERO-2.

SCFA plays an important role in the health of the GIT and their production can be modulated by diet (Abdul Rahim et al., 2019). To evaluate if there are changes in bacterial activities between the different microbial feedings the production of SCFA, BCFA, and other organic acids over time was measured. In this study, the ratio SCFA: BCFA was similar to earlier research of González-Ortiz and colleagues (González-Ortiz et al., 2019). In contrast, the ratio acetate, propionate, and butyrate was not in line with previous research. We found a lower butyrate concentration compared to propionate, whereas most studies show a reversed ratio (Meimandipour et al., 2011; González-Ortiz et al., 2019, 2020). This might be related to the age of the chickens or the type of breed of the chickens. Liao et al. (2020) showed an increase in SCFA and a smaller ratio between propionate and butyrate with increasing age of the chickens. Furthermore, in their research, they used Arbor Acres broiler chicks, whereas we studied Ross 308 broilers (Kers et al., 2019; Liao et al., 2020). When comparing the BCFA and other organic acid production with the SCFA production, a much lower concentration was found, which corresponds with other studies (Qaisrani et al., 2015; González-Ortiz et al., 2020). The delay in the production of the other organic acids, especially for lactate, might have been because lactate was converted in propionate or butyrate before we could have measured the lactate concentration (Duncan et al., 2004; Kovatcheva-Datchary et al., 2009; Venema, 2015).

Both the taxonomic profiles as well as the metabolite production of the CALIMERO-2 samples showed some shifts compared to the *in vivo* situation. These shifts can be related to the new environment the microbiota needs to adapt to, and some factors that are not present in the model, like a mucus layer (Rajilić-Stojanović et al., 2010). The changes in taxonomic profile are seen in most *in vitro* systems (Rajilić-Stojanović et al., 2010; Van Den Abbeele et al., 2010). Nevertheless, although we see a shift in taxonomic profiles in the beginning, the bacterial alpha diversity showed high similarity with the original inoculum after a longer period of fermentation. After an initial reduction after the start of the fermentation an increase in alpha diversity over time was observed and after fermentation for 72 h, the number of bacterial taxa had become more similar to the original inoculum.

For the metabolite production, we cannot simply compare our results to *in vivo* experiments. During *in vivo* experiments, we are limited to random sampling and the cumulative total production of SCFA cannot be measured, despite the possibility to euthanize animals and collect samples from multiple sites of the intestine. Static models are also restricted, because there is an accumulation of metabolites, which might severely influence the metabolic activity of the bacteria, due to inhibition of fermentation at high concentrations. With CALIMERO-2, where metabolites are removed through the dialysis system, we can therefore accurately measure the influence of feed additives on the production of SCFA, and other metabolites.

Another advantage of the CALIMERO-2 system is that experiments are reproducible, and variability usually observed *in vivo* due to interindividual variability, is low because the cecal samples collected are pooled. There are multiple reasons to pool the cecal samples. Firstly, a practical reason, multiple need to be combined, to obtain a sufficient amount of volume to conduct a single experiment. Moreover, to get a good representation of the chicken population, pooling reduces variability between samples. In previous experiments with human microbiota, we observed that, with respect to carbohydrate fermentation, pooling and standardizing of the microbiota from several individuals led to the same microbial activity of the individual microbiota and the pooled inoculum despite differences in microbiota composition (Aguirre et al., 2014). This is due to the vast functional redundancy between microbial taxa, allowing different microorganisms to use the same substrate and produce identical metabolites (Louca et al., 2018). Standardizing the microbiota also allows numerous experiments (in our case close to 100) to be carried out with the same starting microbiota. Furthermore, the model is reproducible, since the system is computer controlled and is run under strict control. In this way, the environmental factors such as pH, temperature and food intake, are the same for each experiment and do not influence the microbiota. In the current study this allowed us to discover that different compositions of the SIEM media did not lead to differences in microbiota composition and activity. Also, the ability to change specific parameters in the system and the large amount of experiments that can be performed with CALIMERO-2 for pre-screening for efficacy of feed interventions, substantially reduces the number of *in vivo* experiments needed for further validation before it can be commercially applied.

## Conclusion

CALIMERO-2 can be used as a digestion system model for the chicken ceca, in which the microbial composition and activity can be maintained in a similar manner to the *in vivo* cecum. Thus, the developed model allows measurements regarding modulation of composition and activity of the chicken cecum microbiota in response to, for instance, feed interventions. The standard growth medium SIEM can be used for experiments within this model.

In future work, CALIMERO-2 can be used to study the effect of several types of dietary substrates, or the effect of for example antibiotics, on the chicken cecal microbiota. In this paper we focused on the bacterial composition, however, the system can also be used to study the complete microbiota, for example the effect of dietary interventions on fungal composition. Furthermore, the digestion system model can also mimic an intestinal disease, like necrotic enteritis caused by the pathogen *Clostridium (C.) perfringens*. In addition to studying the microbiota, the samples obtained from CALIMERO-2 experiments, i.e., fecal waters, can be used for follow-up *in vitro* experiments. *In vitro* cell lines, co-cultures of intestinal cells with immune cells, or organoids mimicking the intestine can be exposed to the fecal waters, to further study effects of products aimed at modulating the gut microbiota on intestinal health.

## Data Availability Statement

The raw sequence data generated during this study are available in the Sequence Read Archive (SRA) repository at the NCBI under accession number PRJNA759368 (available at: https://www.ncbi.nlm.nih.gov/sra/PRJNA759368).

## Author Contributions

MO, FV, AK, and KV contributed to the design of the study. MO and FV collected the samples. MO performed the experiments. MO and KV performed the analysis of data. FV, KV, and AK supervised the work. All authors approved the final version of the manuscript.

## Conflict of Interest

The authors declare that the research was conducted in the absence of any commercial or financial relationships that could be construed as a potential conflict of interest.

## Publisher’s Note

All claims expressed in this article are solely those of the authors and do not necessarily represent those of their affiliated organizations, or those of the publisher, the editors and the reviewers. Any product that may be evaluated in this article, or claim that may be made by its manufacturer, is not guaranteed or endorsed by the publisher.

## Supplementary Material

Supplementary Table 1 **(1)** Microbial feeding composition of SIEM, Modified SIEM-I and Modified SIEM-II. **(2)** Significant difference between bacterial phyla between groups. **(3)** Significant difference between bacterial families between groups.

Supplementary Figure 1 Flow chart of experimental set up of CALIMERO-2. **(A)** Experimental set up for the different sample types, SIEM, Modified SIEM-I and Modified SIEM-II. **(B)** The experimental set up over time. At the times indicated by ^∗^ samples were taken from both the lumen and dialysate.

Supplementary Figure 2 Bacterial alpha diversity. Data are presented as mean (*n* = 2) ± sd. (^∗∗^ *p* < 0.001 and ^∗∗∗^ *p* < 0.0001) **(A)**. Inverse Simpson **(B)**. Gini-Simpsom **(C)**. Fisher **(D)**. coverage.

## References

[B1] Abdul Rahim M. B. H. Chilloux J. Martinez-Gili L. Neves A. L. Myridakis A. Gooderham N. (2019). Diet-induced metabolic changes of the human gut microbiome: importance of short-chain fatty acids, methylamines and indoles. *Acta Diabetol.* 56 493–500. 10.1007/s00592-019-01312-x 30903435 PMC6451719

[B2] Adhikari P. Kiess A. Adhikari R. Jha R. (2020). An approach to alternative strategies to control avian coccidiosis and necrotic enteritis. *J. Appl. Poult. Res.* 29 515–534. 10.1016/j.japr.2019.11.005

[B3] Aguirre M. Ramiro-Garcia J. Koenen M. E. Venema K. (2014). To pool or not to pool? Impact of the use of individual and pooled fecal samples for in vitro fermentation studies. *J. Microbiol. Methods* 107 1–7. 10.1016/j.mimet.2014.08.022 25194233

[B4] Anderson M. J. (2017). *Permutational Multivariate Analysis of Variance (PERMANOVA).* United States: Wiley Online Library. 10.1002/9781118445112.stat07841

[B5] Antonissen G. Eeckhaut V. Van Driessche K. Onrust L. Haesebrouck F. Ducatelle R. (2016). Microbial shifts associated with necrotic enteritis. *Avian Pathol.* 45 308–312. 10.1080/03079457.2016.1152625 26950294

[B6] Attia G. A. Metwally A. E. Beheiry R. R. Farahat M. H. (2021). Effect of a multicarbohydrase supplementation to diets varying in metabolisable energy level on the performance, carcase traits, caecal microbiota, intestinal morphology, and nutrient digestibility in broiler chickens. *Ital. J. Anim. Sci.* 20 215–225. 10.1080/1828051X.2021.1875337

[B7] Bisanz J. E. (2018). *qiime2R: Importing QIIME2 Artifacts and Associated Data into R Sessions.* Available online at: https://github.com/jbisanz/qiime2R

[B8] Bolyen E. E. E. (2019). Reproducible, interactive, scalable and extensible microbiome data science using QIIME 2. *Nature Biotechnology* 37 852–857. 10.1038/s41587-019-0209-9 31341288 PMC7015180

[B9] Borda-Molina D. Seifert J. Camarinha-Silva A. (2018). Current Perspectives of the Chicken Gastrointestinal Tract and Its Microbiome. *Comput. Struct. Biotechnol. J.* 16 131–139. 10.1016/j.csbj.2018.03.002 30026889 PMC6047366

[B10] Callahan B. J. McMurdie P. J. Rosen M. J Han A. W. Johnson AJ. Holmes S. P. (2016). DADA2: high-resolution sample inference from Illumina amplicon data. *Nat. Methods* 13 581–583. 10.1038/nmeth.3869 27214047 PMC4927377

[B11] Chambers J. R. Gong J. (2011). The intestinal microbiota and its modulation for *Salmonella* control in chickens. *Food Res. Int.* 44 3149–3159. 10.1016/j.foodres.2011.08.017

[B12] Corrigan A. De Leeuw M. Penaud-Frézet S. Dimova D. Murphy R. A. (2015). Phylogenetic and functional alterations in bacterial community compositions in broiler ceca as a result of mannan oligosaccharide supplementation. *Appl. Environ. Microbiol.* 81 3460–3470. 10.1128/AEM.04194-14 25769823 PMC4407213

[B13] De Carvalho N. M. Oliveira D. L. Saleh M. A. D. Pintado M. E. Madureira A. R. (2021). Importance of gastrointestinal in vitro models for the poultry industry and feed formulations. *Anim. Feed Sci. Technol.* 271 114730. 10.1016/j.anifeedsci.2020.114730

[B14] De Maesschalck C. Eeckhaut V. Maertens L. De Lange L. Marchal L. Nezer C. (2015). Effects of Xylo-Oligosaccharides on Broiler Chicken Performance and Microbiota. *Appl. Environ. Microbiol.* 81 5880–5888. 10.1128/AEM.01616-15 26092452 PMC4551243

[B15] De Souza C. B. Roeselers G. Troost F. Jonkers D. Koenen M. E. Venema K. (2014). Prebiotic effects of cassava bagasse in TNO’s in vitro model of the colon in lean versus obese microbiota. *J. Funct. Foods* 11 210–220. 10.1016/j.jff.2014.09.019

[B16] Duncan S. H. Louis P. Flint H. J. (2004). Lactate-utilizing bacteria, isolated from human feces, that produce butyrate as a major fermentation product. *Appl. Environ. Microbiol. J.* 70 5810–5817. 10.1128/AEM.70.10.5810-5817.2004 15466518 PMC522113

[B17] Gibson G. R. Probert H. M. Loo J. V. Rastall R. A. Roberfroid M. B. (2004). Dietary modulation of the human colonic microbiota: updating the concept of prebiotics. *Nutr. Res. Rev.* 17 259–275. 10.1079/NRR200479 19079930

[B18] Gong J. Forster R. J. Yu H. Chambers J. R. Wheatcroft R. Sabour P. M. (2002). Molecular analysis of bacterial populations in the ileum of broiler chickens and comparison with bacteria in the cecum. *FEMS Microbiol. Ecol.* 41 171–179. 10.1111/j.1574-6941.2002.tb00978.x 19709251

[B19] González-Ortiz G. Dos Santos T. T. Vienola K. Vartiainen S. Apajalahti J. Bedford M. R. (2019). Response of broiler chickens to xylanase and butyrate supplementation. *Poult. Sci.* 98 3914–3925. 10.3382/ps/pez113 30915461

[B20] González-Ortiz G. Olukosi O. A. Jurgens G. Apajalahti J. Bedford M. R. (2020). Short-chain fatty acids and ceca microbiota profiles in broilers and turkeys in response to diets supplemented with phytase at varying concentrations, with or without xylanase. *Poult. Sci.* 99 2068–2077. 10.1016/j.psj.2019.11.051 32241492 PMC7587645

[B21] Kers J. G. Velkers F. C. Fischer E. A. Hermes G. D. A. Stegeman J. A. Smidt H. (2018). Host and environmental factors affecting the intestinal microbiota in chickens. *Front. Microbiol.* 9:235. 10.3389/fmicb.2018.00235 29503637 PMC5820305

[B22] Kers J. G. Velkers F. C. Fischer E. A. J. Hermes G. D. A. Lamot D. M. Stegeman J. A. (2019). Take care of the environment: housing conditions affect the interplay of nutritional interventions and intestinal microbiota in broiler chickens. *Anim. Microb.* 1:10. 10.1186/s42523-019-0009-z 33499936 PMC7807522

[B23] Kovatcheva-Datchary P. Egert M. Maathuis A. Rajiliæ-Stojanoviæ M. De Graaf A. A. Smidt H. (2009). Linking phylogenetic identities of bacteria to starch fermentation in an in vitro model of the large intestine by RNA-based stable isotope probing. *Environ. Microbiol.* 11 914–926. 10.1111/j.1462-2920.2008.01815.x 19128319

[B24] Liao X. Shao Y. Sun G. Yang Y. Zhang L. Guo Y. (2020). The relationship among gut microbiota, short-chain fatty acids, and intestinal morphology of growing and healthy broilers. *Poult. Sci.* 99 5883–5895. 10.1016/j.psj.2020.08.033 33142506 PMC7647869

[B25] Long C. De Vries S. Venema K. (2020). Polysaccharide source altered ecological network, functional profile, and short-chain fatty acid production in a porcine gut microbiota. *Benef. Microbes* 11 591–610. 10.3920/BM2020.0006 32936008

[B26] Louca S. Polz M. F. Mazel F. Albright M. B. N. Huber J. A. O’connor M. I. (2018). Function and functional redundancy in microbial systems. *Nat. Ecol. Evol.* 2 936–943. 10.1038/s41559-018-0519-1 29662222

[B27] Lu M. Li R. W. Zhao H. Yan X. Lillehoj H. S. Sun Z. (2020). Effects of Eimeria maxima and Clostridium perfringens infections on cecal microbial composition and the possible correlation with body weight gain in broiler chickens. *Res. Vet. Sci.* 132 142–149. 10.1016/j.rvsc.2020.05.013 32575030

[B28] Maathuis A. Hoffman A. Evans A. Sanders L. Venema K. (2009). The effect of the undigested fraction of maize products on the activity and composition of the microbiota determined in a dynamic in vitro model of the human proximal large intestine. *J. Am. Coll. Nutr.* 28 657–666. 10.1080/07315724.2009.10719798 20516265

[B29] Mabelebele M. Alabi O. Ngambi J. Norris D. Ginindza M. (2014). Comparison of gastrointestinal tracts and pH values of digestive organs of Ross 308 broiler and indigenous Venda chickens fed the same diet. *Asian J. Anim. Vet. Adv.* 9 71–76. 10.3923/ajava.2014.71.76

[B30] Meimandipour A. Soleimanifarjam A. Azhar K. Hair-Bejo M. Shuhaimi M. Nateghi L. (2011). Age effects on short chain fatty acids concentrations and pH values in the gastrointestinal tract of broiler chickens. *Arch. Fur Geflugelkunde* 75 164–168.

[B31] Minekus M. Smeets-Peeters M. Bernalier A. Marol-Bonnin S. Havenaar R. Marteau P. (1999). A computer-controlled system to simulate conditions of the large intestine with peristaltic mixing, water absorption and absorption of fermentation products. *Appl. Microbiol. Biotechnol.* 53 108–114. 10.1007/s002530051622 10645630

[B32] Oakley B. B. Kogut M. H. (2016). Spatial and Temporal Changes in the Broiler Chicken Cecal and Fecal Microbiomes and Correlations of Bacterial Taxa with Cytokine Gene Expression. *Front. Vet. Sci.* 3:11. 10.3389/fvets.2016.00011 26925404 PMC4759570

[B33] Ocejo M. Oporto B. Hurtado A. (2019). 16S rRNA amplicon sequencing characterization of caecal microbiome composition of broilers and free-range slow-growing chickens throughout their productive lifespan. *Sci. Rep.* 9:2506. 10.1038/s41598-019-39323-x 30792439 PMC6385345

[B34] Parks D. H. Tyson G. W. Hugenholtz P. Beiko R. G. (2014). STAMP: statistical analysis of taxonomic and functional profiles. *Bioinformatics* 30 3123–3124. 10.1093/bioinformatics/btu494 25061070 PMC4609014

[B35] Qaisrani S. N. Van Krimpen M. M. Kwakkel R. P. Verstegen M. W. A. Hendriks W. H. (2015). Diet structure, butyric acid, and fermentable carbohydrates influence growth performance, gut morphology, and cecal fermentation characteristics in broilers. *Poult. Sci.* 94 2152–2164. 10.3382/ps/pev003 26175052 PMC4988549

[B36] Rajilić-Stojanović M. Maathuis A. Heilig H. G. H. J. Venema K. De Vos W. M. Smidt H. (2010). Evaluating the microbial diversity of an in vitro model of the human large intestine by phylogenetic microarray analysis. *Microbiology* 156 3270–3281. 10.1099/mic.0.042044-0 20847013

[B37] R Core Team (2020). *R: A Language and Environment for Statistical Computing.* Vienna: R Foundation for Statistical Computing.

[B38] Round J. L. Mazmanian S. K. (2009). The gut microbiota shapes intestinal immune responses during health and disease. *Nat. Rev. Immunol.* 9 313–323. 10.1038/nri2515 19343057 PMC4095778

[B39] Rychlik I. (2020). Composition and Function of Chicken Gut Microbiota. *Animals* 10:103. 10.3390/ani10010103 31936291 PMC7022619

[B40] Scott K. P. Gratz S. W. Sheridan P. O. Flint H. J. Duncan S. H. (2013). The influence of diet on the gut microbiota. *Pharmacol. Res.* 69 52–60. 10.1016/j.phrs.2012.10.020 23147033

[B41] Svihus B. (2014). Function of the digestive system1 1Presented as a part of the Informal Nutrition Symposium “From Research Measurements to Application: bridging the Gap” at the Poultry Science Association’s annual meeting in San Diego, California, July 22–25, 2013. *J. Appl. Poult. Res.* 23 306–314. 10.3382/japr.2014-00937

[B42] Van Den Abbeele P. Grootaert C. Marzorati M. Possemiers S. Verstraete W. Gérard P. (2010). Microbial community development in a dynamic gut model is reproducible, colon region specific, and selective for Bacteroidetes and Clostridium cluster IX. *Appl. Environ. Microbiol.* 76 5237–5246. 10.1128/AEM.00759-10 20562281 PMC2916472

[B43] Van Der Wielen P. W. Biesterveld S. Notermans S. Hofstra H. Urlings B. A. Van Knapen F. (2000). Role of volatile fatty acids in development of the cecal microflora in broiler chickens during growth. *Appl. Environ. Microbiol.* 66 2536–2540. 10.1128/AEM.66.6.2536-2540.2000 10831435 PMC110578

[B44] Van Der Wielen T. Boon N. Possemiers S. Jacobs H. Verstraete W. (2004). Prebiotic effects of chicory inulin in the simulator of the human intestinal microbial ecosystem. *FEMS Microbiol. Ecol.* 51 143–153. 10.1016/j.femsec.2004.07.014 16329863

[B45] Venema K. (2015). “The TNO In Vitro Model of the Colon (TIM-2)” in *The Impact of Food Bioactives on Health: in Vitro and ex Vivo Models.* eds Verhoeckx K. Cotter P. López-Expósito I. Kleiveland C. Lea T. Mackie A.. et al. (Berlin: Springer International Publishing). 29787064

[B46] Venema K. Nuenen M. V. Smeets-Peeters M. Minekus M. Havenaar R. (2000). TNO’s in vitro large intestinal model: an excellent screening tool for functional food and pharmaceutical research. *Ernährung* 24 558–564.

[B47] Venema K. Van Nuenen M. H. M. C. Van Den Heuvel E. G. Pool W. Van Der Vossen J. M. B. M. (2003). The Effect of Lactulose on the Composition of the Intestinal Microbiota and Short-chain Fatty Acid Production in Human Volunteers and a Computer-controlled Model of the Proximal Large Intestine. *Microbial. Ecol. Health Dis.* 15 94–105. 10.1080/08910600310019895

[B48] Xiao Y. Xiang Y. Zhou W. Chen J. Li K. Yang H. (2017). Microbial community mapping in intestinal tract of broiler chicken. *Poult. Sci.* 96 1387–1393. 10.3382/ps/pew372 28339527

[B49] Yadav S. Jha R. (2019). Strategies to modulate the intestinal microbiota and their effects on nutrient utilization, performance, and health of poultry. *J. Anim. Sci. Biotechnol.* 10:2. 10.1186/s40104-018-0310-9 30651986 PMC6332572

[B50] Zhu X. Y. Zhong T. Pandya Y. Joerger R. D. (2002). 16S rRNA-Based Analysis of Microbiota from the Cecum of Broiler Chickens. *Appl. Environ. Microbiol.* 68 124–137. 10.1128/AEM.68.1.124-137.2002 11772618 PMC126585

