# Supplementary material for: Development of the in vitro Cecal Chicken ALIMEntary tRact mOdel-2 to Study Microbiota Composition and Function
Source: Front Microbiol. 2021 Oct 11;12:726447. doi: 10.3389/fmicb.2021.726447 (PMC8542841; doi:10.3389/fmicb.2021.726447)
Supplement: Supplementary Figure 1 — Flow chart of experimental set up of CALIMERO-2. (A) Experimental set up for the different sample types, SIEM, Modified SIEM-I and Modified SIEM-II. (B) The experimental set up over time. At the times indicated by ∗ samples were taken from both the lumen and dialysate. [file Image_1.pdf]

Supplementary figures and tables_CALIMERO-2 manuscript

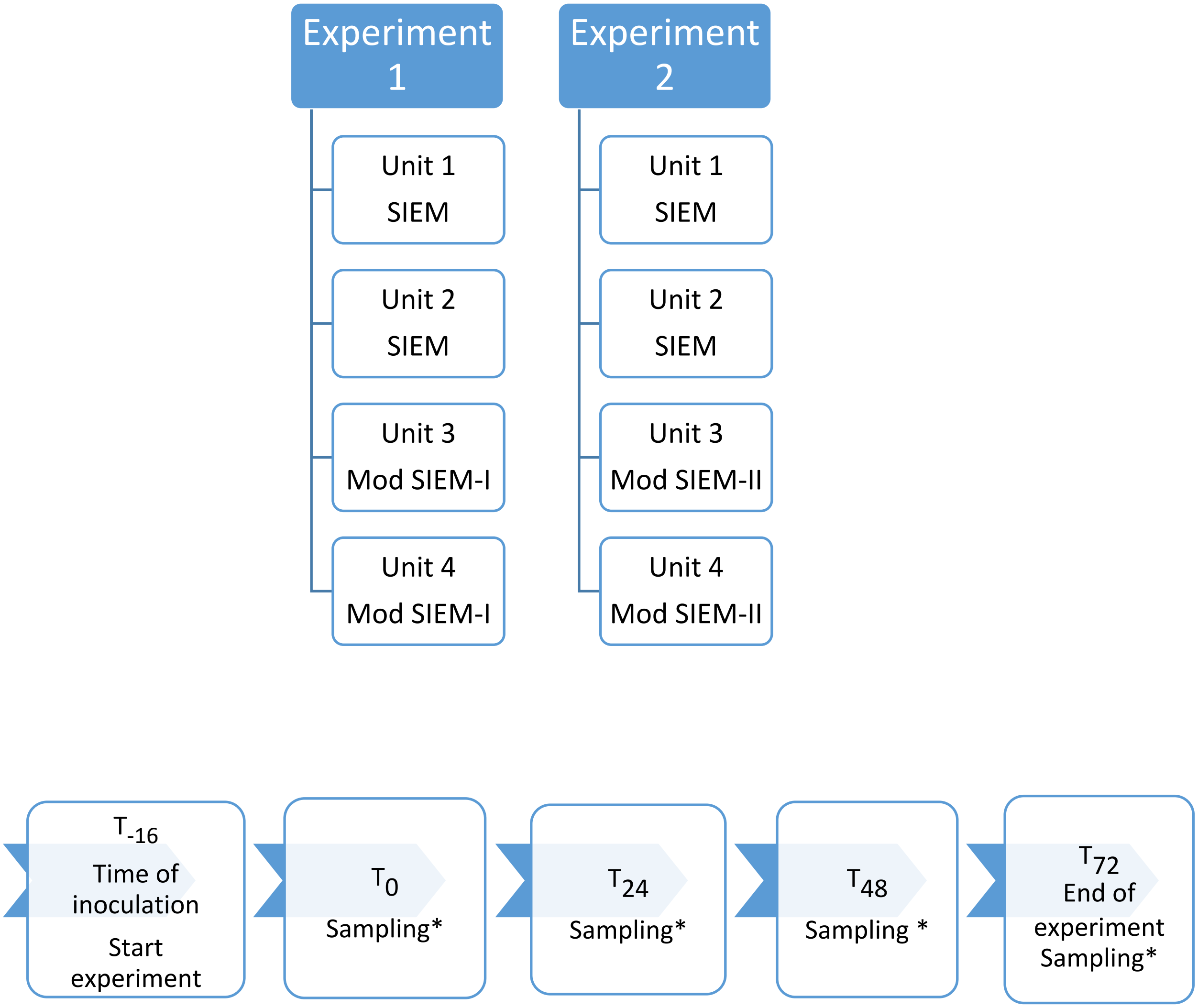

Supplementary Figure 1. Flow chart of experimental set up of CALIMERO-2. A. Experimental set up for the different sample types, SIEM, Modified SIEM-I and Modified SIEM-II. **B.** The experimental set up over time. At the times indicated by * samples were taken from both the lumen and dialysate.
