# Supplementary figures and images for: Development of the in vitro Cecal Chicken ALIMEntary tRact mOdel-2 to Study Microbiota Composition and Function
Source: Front Microbiol. 2021 Oct 11;12:726447. doi: 10.3389/fmicb.2021.726447 (PMC8542841; doi:10.3389/fmicb.2021.726447)

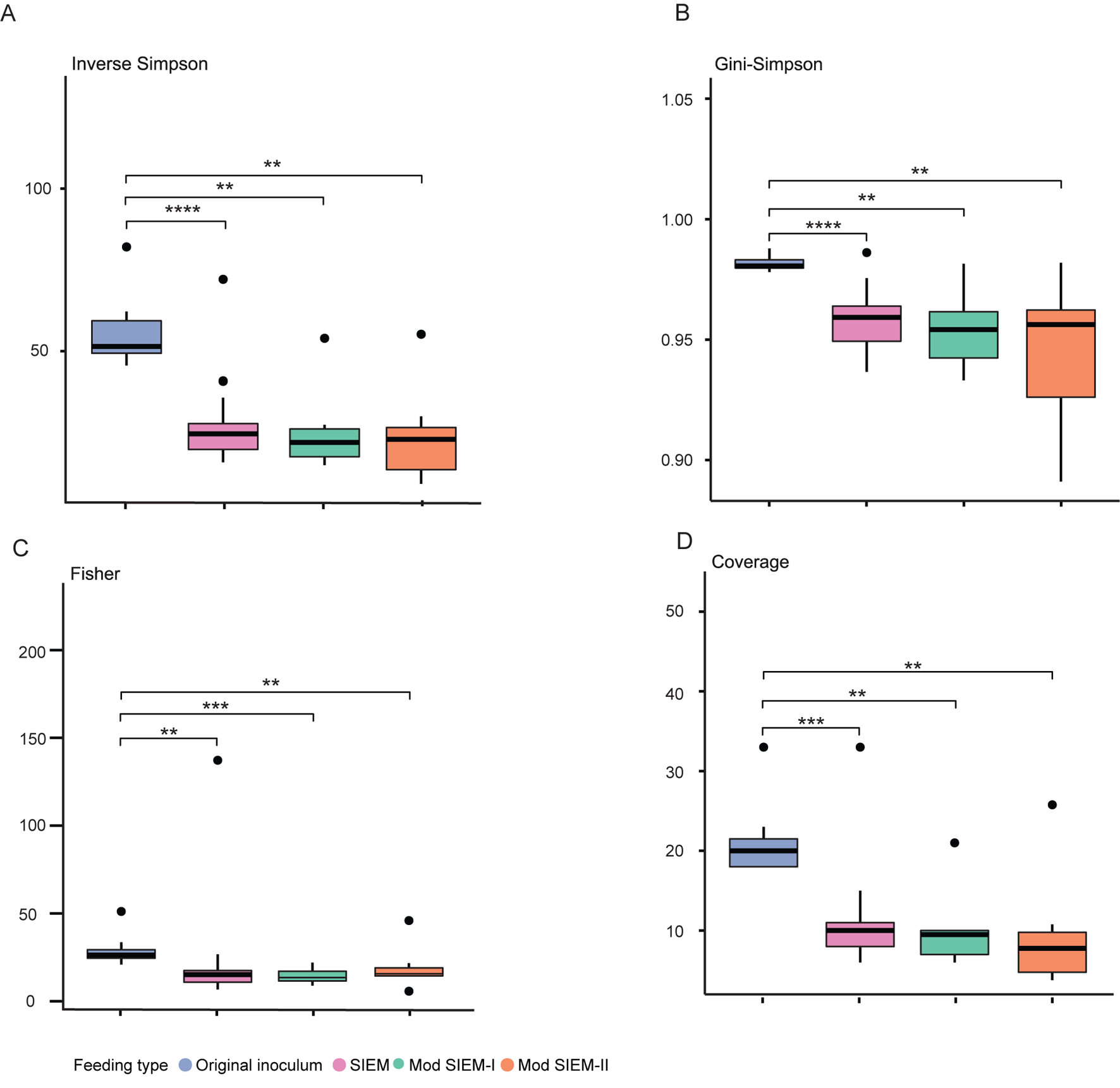

Supplement: Supplementary Figure 2 — Bacterial alpha diversity. Data are presented as mean (n = 2) ± sd. (∗∗ p < 0.001 and ∗∗∗ p < 0.0001) (A). Inverse Simpson (B). Gini-Simpsom (C). Fisher (D). coverage. [file Image_2.tif]
